# Mechanical Design and Control Strategy for Hip Joint Power Assisting

**DOI:** 10.1155/2018/9712926

**Published:** 2018-08-15

**Authors:** Wenyuan Liang

**Affiliations:** ^1^College of Engineering, Peking University, Beijing 100870, China; ^2^National Research Center for Rehabilitation Technical Aids, Beijing 100176, China

## Abstract

The basic requirements for mechanical design and control strategy are adapting to human joint movements and building an interaction model between human and robot. In this paper, a 3-UPS parallel mechanism is adopted to realize that the instantaneous rotation center of the assistive system coincides with human joint movement center, and a force sensory system is used to detect human movement intention and build the modeling of control strategy based on the interactive force. Then, based on the constructed experimental platform, the feasibility of movement intention detection and power assisting are verified through the experimental results.

## 1. Introduction

Assistive robot, also called as a powered exoskeleton robot, is a special robot that aims at improving the capability and efficiency of users. The assistive robots have been developed to be used for human limb strength [1], neurorehabilitation [2], or movement assistance [3]. The challenges for current research of assistive robot include the followings: how to design an assistive robot to adapt human movement, how to obtain human movement intention to promote the human-machine interaction, and how to control the robot to provide an effective assistance.

The design of an assistive robot should consider the physical structure of the joint and muscle to support the body weight during movements. Hip joint, as one important part of the human lower limb, is considered as a spherical joint with three DoFs. Some studies have designed kinds of assistive robots for hip joint power assisting. For example, BLEEX [4] and NAEIES [5], with an anthropomorphic design, are active on the hip flexion/extension (f/e) and abduction/adduction (a/a) and passive on the hip intra/extrarotation.

Movement assistance process is also called as human-machine interaction, which requires a strong synergy between the user and the assistive robot. During the movements, the user's joint and muscle generate the activation, and then the assistive robot provides the user's joints with supplemental torques. In the human-machine interaction process, obtaining the human movement intention is fundamental for controlling the assistive robot. The sensory systems are used to detect users' movement intention or muscular activities during the process of human-machine interaction, which could be detected directly by measuring EMG, interaction force sensors, gait information, and/or even EEG. HAL is developed to aid people moving and executing daily-life activities. In [6], HAL mainly supports the persons with motion difficulties but whose EMG signals can still be detected from the muscle. NAEIES [5] is developed for helping the user carry heavy loads, where the human-machine interaction force is measured by multiaxis force/torque sensors. AAFO, developed by Yonsei University, uses four force sensors to detect the gait events [7].

Based on the movement intention detection, the assistive controllers would determine the supplemental torque provided by the assistive robot. The controllers should ensure that the provided assistive torques are coherent with human joint and muscle own activation. In [8], the robot is activated with an EMG-based controller, where the desired trajectories in the actuators are related to the processed EMG signals of the selected muscles. In [9, 10], the actuators are driven based on the walking gait information. Model-based control [11–13] needs to build the kinematic or dynamic model for the human-machine interaction model. Since it is difficult to obtain the inertia parameters of the human limb, some model parameters need to be estimated.

The interaction processes are various among different users. The signal processing and interaction modeling are time-consuming, especially for the methods based on EMG [14], EEG [15, 16], and gait information. Compared to the abovementioned methods, the method based on the force sensor is a better choice, since different objects difference will have less influence on the model of interactive force between human body and assistive robot. Additionally, aiming to provide power assisting for the users who do not lose muscle strength completely, the interactive force sensory system is considered as the intention detection method in this paper.

Though the characteristics of the joints and limbs differ significantly in different users, the control process is usually based on the sensory systems, which are fundamental for the control strategy. The key of the assistive robot is to respond to human movement almost without any delay. In order to control the assistive robot with good performance on providing power assisting, the controller should be good at dealing with the human-machine interaction. Considering the interaction is measured based on the force sensor, a force-based compliance controller is proposed in this paper.

This paper is organized as follows: the second section will show the hip joint assistive robot structural design and kinematic model, and the assistive robot control based on the human-machine interactive force is designed; in the third section, the principle for using the force sensor to detect human movement intention is discussed, and the assistive results based on the compliance control is included; and the last section is the conclusion.

## 2. Materials and Methods

### 2.1. Structure Design and Modeling

#### 2.1.1. Mechanical Structure Design

The structure design for the hip joint assistive robot should consider the following requirements:

1. Hip joint is considered as a spherical joint of 3 DoFs, which are f/e DoF, a/a DoF, and intra/extrarotation DoF.
2. The assistive robot movement can cover all the three DoFs.
3. The assistive robot can kinematically adapt to the movement of the hip joint.

**Figure 1:**
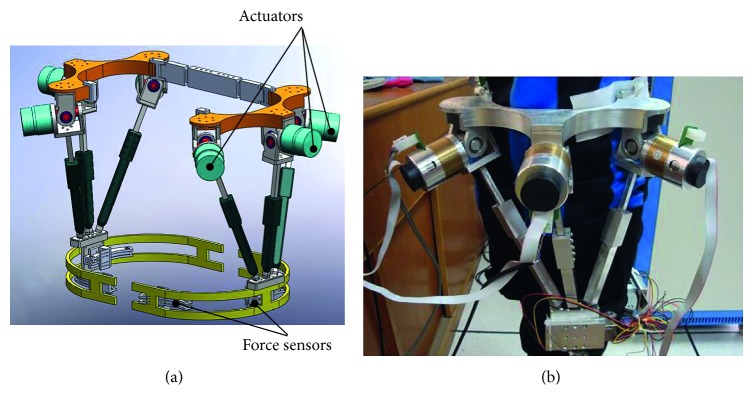
(a) CAD model; (b) hip joint power assisting robot.

**Figure 2:**
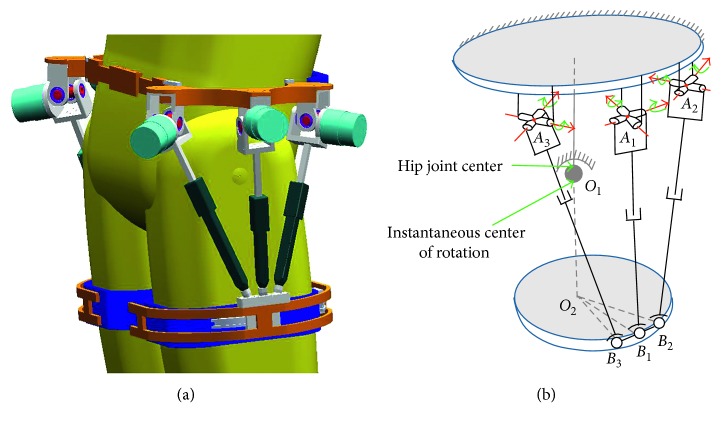
A parallel mechanism for hip joint power assisting.

Then, a 3-UPS parallel mechanical assistive robot can meet the above requirements. As shown in Figures 1 and 2, while the human wears the hip joint assistive robot, the physical model can be simplified as a 3-UPS/1-S model. In this model, the mechanical instantaneous center of rotation is the hip joint center.

In this way, a 3-UPS parallel mechanism can realize 3-DoFs motions without disturbing the human hip joint movement.

#### 2.1.2. Assistive Robot Modeling

**Figure 3:**
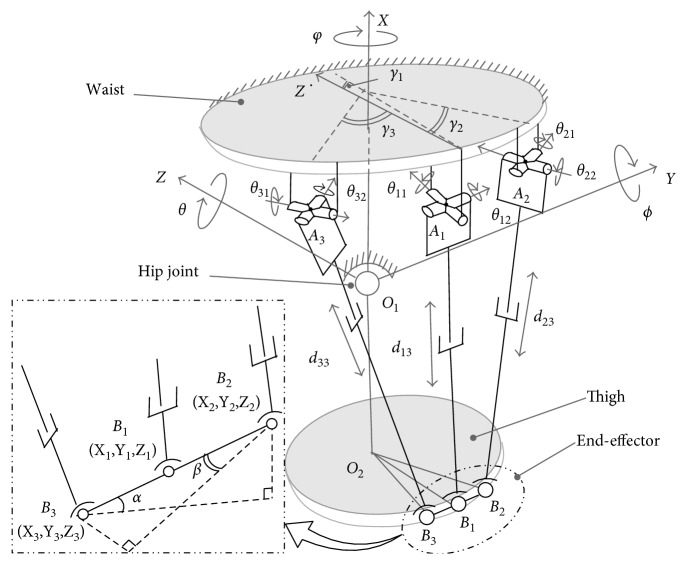
A simplified model for the hip joint assisting robot.

As shown in Figure 3, assistive mechanism drives the thigh bandage (which are composed of points B_1_, B_2_, and B_3_) to provide assistance for the human thigh. Hence, the bandage is considered as the end-effector of the assistive robot. The end-effector has three DoFs, where parameters $\left({\dot{Z}}_{1},\dot{\alpha },\;\;\mathrm{and}\;\;\dot{\beta }\right)$ (Figure 3) can describe end-effector's movements. ${\dot{Z}}_{1}$ and $\dot{\alpha }$ can describe end-effector's motions of abduction/adduction and flexion/extension, respectively; thigh's rotation velocity along the longitudinal axis is defined as $\dot{\beta }$.

By considering the installation positions of motors, *θ*_11_, *θ*_22_, and *θ*_32_ are selected as active joints. These three active joints are driven with the brushless DC motors (Maxon EC-45 flat series, Figure 1). The Jacobian representing the velocity relation between end-effector and active joints is given as follows [17]:

(1) $$\begin{array}{c} {\left[{\dot{Z}}_{1}\;\dot{\alpha }\;\dot{\beta }\right]}^{T}=J\cdot {\left[{\dot{\theta }}_{11}\;{\dot{\theta }}_{22}\;{\dot{\theta }}_{32}\right]}^{T}, \end{array}$$

where [*∗*]^*T*^ is the transpose of the matrix [*∗*]. Since thigh muscle's movement intention would finally act on end-effector's movement, $\left[\begin{array}{ccc} {\dot{Z}}_{1} & \dot{\alpha } & \dot{\beta } \end{array}\right]$ can represent thigh muscle's movement intention indirectly. Here, Jacobian **J** describes the velocity relation between thigh's movement intention and actuators. Through (1), we can correspond muscle's movement intention with an active actuator.

In this case, according to the force feedback between assisted limb and assistive robot, the expected end-effector velocity of the human thigh with the controller can be generated. And then with the inverse compute based on (1), the expected velocities of actuators can be obtained.

### 2.2. Controller Design

The assistive mechanism is a system that provides power assisting through human-machine interaction. In the process of interaction, the key is to obtain human movement intention. Then further, in order to provide power assist, it is needed to develop a control strategy based on the movement intention.

#### 2.2.1. Movement Intention Detection Based on Interactive Force Sensor

**Figure 4:**
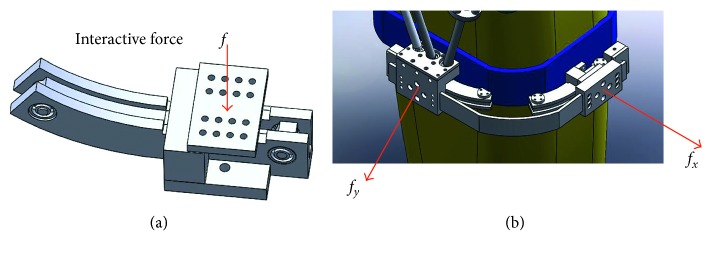
Human movement intention detection based on the force sensor. (a) Force sensor. (b) Interactive force detection.

In this paper, two one-dimensional force sensors (Figure 4) are used to detect human movement intention on the motions of f/e and a/a. The force sensor shown in Figure 4 is of high sensitivity. Thereby, it can react quickly to the human-machine interaction. The detection force *f*_*x*_ is in the sagittal plane and mainly used to detect the movement intention of extension/flexion. The detection force *f*_*y*_ is in the coronal plane and mainly used to detect the movement intention of adduction/abduction. In this paper, we mainly focus on providing assistance for the movement of extension/flexion and adduction/abduction.

#### 2.2.2. Compliant Control for Power Assisting

**Figure 5:**
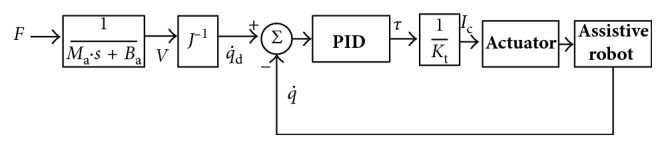
Control strategy based on compliance control.

By online estimating and planning the assistive torque, the proposed compliance controller (shown in Figure 5) is aiming to follow human movement intention and transfer the desired assistive torque to the user's leg effectively.

The controller is motivated below by considering the traditional force control model [18]:

(2) $$\begin{array}{c} \frac{\dot{x}\left(s\right)}{f\left(s\right)}=R\left(s\right)=\frac{1}{M_{\text{a}}\cdot s+B_{\text{a}}+\left(D_{\text{a}}/s\right)}, \end{array}$$

where (2) represents the interactive force control model with the Laplace transform. *M*_a_, *B*_a_, and *D*_a_ are the inertia, damping, and spring coefficients, respectively.

While (2) is written as the time-domain form, it may have three kinds of expression. In this paper, we consider the reference commanded position is given and unchanged. Then, we will have

(3) $$\begin{array}{c} M_{\text{a}}\cdot \ddot{X}+B_{\text{a}}\cdot \dot{X}+D_{\text{a}}\cdot \left(X-X_{\text{d}}\right)=F, \end{array}$$

where *X* represents the variables in the Cartesian space, *X*_d_ represents the reference commanded position, and *F* represents the expected interaction force. In this paper, the expected interaction force is equal to the actual interaction force.

During the assistive process, the assistive robot is expected to follow human movement almost without any delay. That is to say, the assistive robot should not lead front or fall behind the human current position too much. Therefore, the reference commanded position is given as the human current position, *X*_d_=*X*. Then, (3) is rewritten as follows:

(4) $$\begin{array}{c} M_{\text{a}}\cdot \ddot{X}+B_{\text{a}}\cdot \dot{X}=F. \end{array}$$

Equation (4) also has another meaning: in (3), when the spring factor (*D*_a_) in the interaction model is smaller, the compliance effect is better; hence, when the spring factor is too small to ignore, we can set *D*_a_=0, and then we can also have the same expression as (4). Under the model shown in (4), the controller could have a better compliance, and then the assistive robot could follow human movement better.

For (4), its Laplace transform can be written as

(5) $$\begin{array}{c} \frac{V\left(s\right)}{f\left(s\right)}=\frac{1}{M_{\text{a}}\cdot s+B_{\text{a}}}, \end{array}$$

where $V\left(s\right)=\dot{x}\left(s\right)$. In the time domain, (5) is expressed as

(6) $$\begin{array}{c} \frac{M_{\text{a}}}{B_{\text{a}}}\cdot \frac{dV\left(t\right)}{dt}+V\left(t\right)=\frac{1}{B_{\text{a}}}\cdot F\left(t\right). \end{array}$$

By considering the discrete form, (6) is written as follows:

(7) $$\begin{array}{c} \frac{M_{\text{a}}}{B_{\text{a}}}\cdot \frac{V_{n}\left(t\right)-V_{n-1}\left(t\right)}{T}+V_{n}\left(t\right)=\frac{1}{B_{\text{a}}}\cdot F_{n}\left(t\right), \end{array}$$

where *T* is the sampling cycle. The label *n* represents the current sampling time, and the label *n* − 1 represents the last one sampling time.

Thereby, the expected commanded velocity of the assistive robot end-effector, *V*_*n*_(*t*), is calculated by the following expression:

(8) $$\begin{array}{c} V_{n}\left(t\right)=\frac{T}{M_{\text{a}}+B_{\text{a}}\cdot T}\cdot F_{n}\left(t\right)+\frac{M_{\text{a}}}{M_{\text{a}}+B_{\text{a}}\cdot T}\cdot V_{n-1}\left(t\right). \end{array}$$

**Figure 6:**
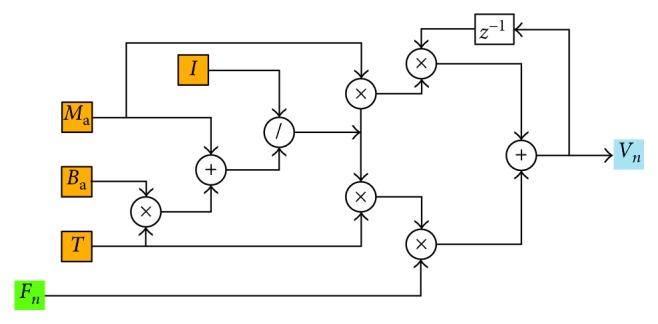
Solution of expected commanded velocity.

In (8), the expected commanded velocity *V*_*n*_(*t*) is related to the current interactive force *F*_*n*_(*t*) and the previous velocity *V*_*n*−1_(*t*). The computation process can be described as Figure 6.

Combined with (1), we can obtain the desired joint velocity ${\dot{q}}_{\text{d}}$ as follows:

(9) $$\begin{array}{c} {\dot{q}}_{\text{d}}=J^{-1}\cdot V_{n}\left(t\right). \end{array}$$

By considering the real-time velocity feedback, $\dot{q}$, and PID control, then the final torque command for each joint or the actuator is *τ*. In Figure 5, *K*_t_ is the torque coefficient for the actuators, and then the torque command is transferred into the current command for each motor.

## 3. Results and Discussion

### 3.1. Feasibility of Movement Intention Detection Based on Interactive Force Sensory System

Human joint movement is composed of three stages that address the following issues: (1) human brain generates the movement intention, and simultaneously, the pallium would generate the relevant movement nerve signals; (2) the nerve signals would transmit from the brain to the agonist's muscle corresponding the neuron, and then the neuron will induce the agonist's muscle to activate; and (3) when the agonist's muscle activates enough, the muscle would finally bring the joint to move. Among these three stages, the first stage is happening in the brain, which may be detected by the EEG; in the second stage, the joint is not moving, but the agonist's muscle is activated which can be detected by the EMG or force sensor; and in the last stage, the joint is moving under the activation of agonist's muscle, and its movement trajectory can be detected by the encoder. The sequence of these three stages is denoted by a time label, where *T*_A_ is the time cost from the beginning of stage 1 to the end of stage 2, *T*_B_ is the time cost from the beginning of stage 2 to the end of stage 3, and *T*_C_ is the time cost from the beginning of stage 1 to the end of stage 3.

According to the current literature, the transmission of nerve signals from the brain to the agonist's muscle differs according to different movement types. When the movement is performed in response to an external stimulus, the same neuron may discharge hundreds of milliseconds before a slow and accurate movement of small amplitude or only 60∼100 ms (*T*_A_) before a ballistic movement [19]. The ballistic movement can be detected by EMG or force sensor. Subsequently, the triggered movements could be executed to act with joint movements in 80∼120 ms (*T*_C_) [20].

The time cost of the human joint ready to move, denoted as (*T*_B_=*T*_C_ − *T*_A_), is about 20 ms. *T*_B_ means the time cost from the time node that the nerve signals induce the muscle activation to the time node that the joint starts to move. The force sensor reaction should be reacted quickly in stage 3, where its time cost, denoted as *T*_D_, should be in the range *T*_D_ ∈ (0, *T*_B_]. In our project, the force sensor can react opposite to the pressure in 1 ms. Thereby, when the controller obtains the human movement intention from the force sensor reaction, the assistive mechanism should act in 19 ms since it detects the movement intention.

**Figure 7:**
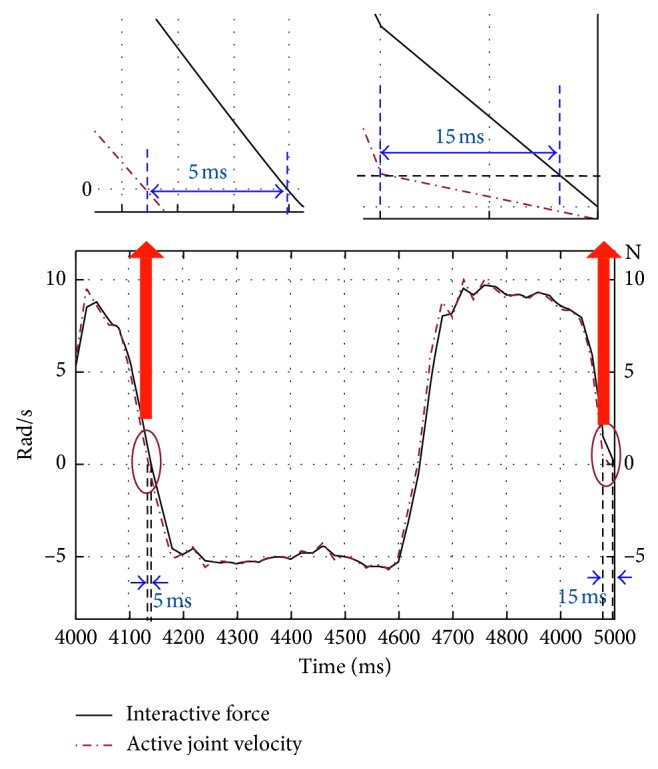
Human movement intention based on interactive force sensor reaction.

In Figure 7, the reaction force curve is obtained by the interactive force sensor, where the curve represents the movement intention of the agonist's muscle; the actuator acting trajectory belongs to the active joint of *θ*_11_. It is found that the actuator acting trajectory is little lagging behind than the reaction force curve. However, the partial enlarged drawings show that, after obtaining the movement intention, the assistive robot can act in 5∼15 ms, which is smaller than 19 ms.

In short, the interactive force sensor-based movement intention detection method adopted in our project can ensure the assistive robot follows human joint movement without any delay.

### 3.2. Assistive Robot Provides Power Assisting Based on Force Sensor

**Figure 8:**
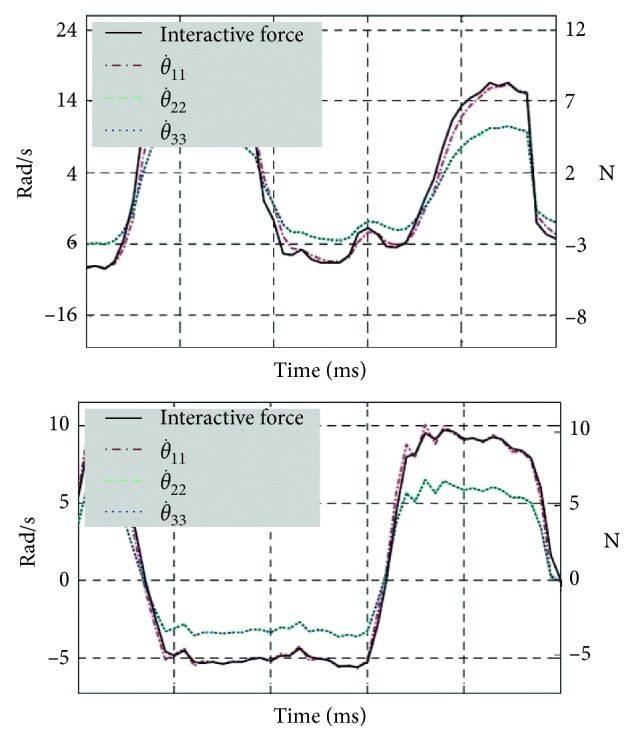
Force sensor interactive trajectories and actuators' tracking trajectories.

Figure 8 shows that the assistive robot provides power assisting while the human joint does the active movement. It can be found out that the active actuators' acting trajectories can follow the interactive force trajectories well.

In Figure 8, it consists of double meanings. First, the interactive force curves, which represent the joint movement intention, are smooth without too much sharp jitter. This characteristic means that the interactive force sensors can detect human movement intention exactly and without delay, and then the force information can be used as the input for the control. Second, based on the interactive force information, the compliance controller in this paper can control the assistive robot to follow human movement almost without delay. It is also found that the actuators' trajectories are smooth, which means that the interaction between human and the assistive robot is with well compliance.

In short, it means that force sensors can obtain the human movement intention quickly and exactly. And then, the assistive robot can also follow human joint movement quickly without delay. In this way, these experiment results ensure the assistive robot can provide power assistance for the user.

## 4. Conclusions

In this paper, the mechanical design and control strategy for a parallel hip joint assistive robot are proposed. The mechanical design is based on a 3-UPS parallel structure. The controller design is based on compliance control with interactive force sensors. The experiment results show that the interactive force-based movement intention detection is available, and the compliance controller also has a good performance in following human movements by providing power assist.

In the future work, it needs to address three issues: (1) in this paper, the coefficients of *M*_a_ and *B*_a_ are determined by many times of trials. We would like to use much adaptive optimized method to determine these two coefficients. (2) The compliance proposed in this paper has a good performance during the human movement process. However, as shown in the formula of (8), the controller could not have a good performance while the interactive force equals to zero. (3) The assistive effect is needed to be assessed via the EMG to detect the activation difference of the agonist's muscle with and without assisting.
